# *Fusarium pseudograminearum* biomass and toxin accumulation in wheat tissues with and without Fusarium crown rot symptoms

**DOI:** 10.3389/fpls.2024.1356723

**Published:** 2024-05-21

**Authors:** Fei Xu, Ruijie Shi, Lulu Liu, Shufang Li, Junmei Wang, Zihang Han, Wei Liu, Hongqi Wang, Jihong Liu, Jieru Fan, Aolin Wang, Chaohong Feng, Yuli Song, Yilin Zhou, Xiangming Xu

**Affiliations:** ^1^ State Key Laboratory for Biology of Plant Diseases and Insect Pests, Institute of Plant Protection, Chinese Academy of Agricultural Sciences, Beijing, China; ^2^ Institute of Plant Protection, Henan Academy of Agricultural Sciences, Zhengzhou, China; ^3^ Key Laboratory of Integrated Pest Management on Crops in Southern Part of North China, Ministry of Agriculture and Rural Affairs of the People’s Republic of China, Zhengzhou, China; ^4^ Institute of Quality Standard and Testing Technology for Agro-products, Henan Academy of Agricultural Sciences, Zhengzhou, China; ^5^ Key Laboratory of Grain Quality and Safety and Testing Henan Province, Henan Academy of Agricultural Sciences, Zhengzhou, China; ^6^ National Institute of Agricultural Botany, East Malling Research, East Malling, Kent, United Kingdom

**Keywords:** Fusarium crown rot (FCR), Fusarium pseudograminearum, Deoxynivalenol (DON), F. pseudograminearum biomass, mycotoxin accumulation

## Abstract

Fusarium crown rot (FCR) is an important and devastating disease of wheat (*Triticum aestivum*) caused by the fungus *Fusarium pseudograminearum* and related pathogens. Using two distinct susceptible cultivars, we investigated the isolation frequencies of *F. pseudograminearum* and quantified its biomass accumulation and the levels of the associated toxins deoxynivalenol (DON) and DON-3-glucoside (D3G) in inoculated field-grown wheat plants. We detected *F. pseudograminearum* in stem, peduncle, rachis, and husk tissues, but not in grains, whereas DON and D3G accumulated in stem, rachis, husk, and grain tissues. Disease severity was positively correlated with the frequency of pathogen isolation, *F. pseudograminearum* biomass, and mycotoxin levels. The amount of *F. pseudograminearum* biomass and mycotoxin contents in asymptomatic tissue of diseased plants were associated with the distance of the tissue from the diseased internode and the disease severity of the plant. Thus, apparently healthy tissue may harbor *F. pseudograminearum* and contain associated mycotoxins. This research helps clarify the relationship between *F. pseudograminearum* occurrence, *F. pseudograminearum* biomass, and mycotoxin accumulation in tissues of susceptible wheat cultivars with or without disease symptoms, providing information that can lead to more effective control measures.

## 1 Introduction

Fusarium crown rot (FCR) is a severe disease that infects wheat (*Triticum aestivum*) in many arid and semi-arid regions worldwide, including portions of the Pacific Northwest of USA (Idaho, Oregon, and Washington), Australia, and the North China Plain (NCP) (Smiley and Patterson, 1996; Burgess et al., 2001; Backhouse et al., 2004; Smiley et al., 2005a; Xu et al., 2018). FCR is primarily caused by the fungus *Fusarium pseudograminearum* (*Fpg*), and to a lesser extent by *F. culmorum* and *F. graminearum* (Chakraborty et al., 2006). In China, FCR caused by *Fpg* was first reported in 2012 in Henan Province (Li et al., 2012), and has since become increasingly common and severe in the NCP (Xu et al., 2018; Deng et al., 2020). In the field, *Fpg* diminished winter wheat yields by as much as 35–51%, with an average drop in yield of nearly 10% for 13 monitored fields (Smiley et al., 2005a; Xu et al., 2016). FCR has caused even greater yield losses in some areas of Australia (Chakraborty et al., 2006). The occurrence of FCR not only causes yield loss, but also results in the accumulation of pathogen biomass and mycotoxins in the stem of plants, providing a reservoir for additional infection and a threat to human and livestock health (Moretti et al., 2014; Knight et al., 2017; Beccari et al., 2018).

The FCR pathogens infect the root or stem base of wheat and spread systemically as the plant grows (Knight and Sutherland, 2015; 2016; Beccari et al., 2018; Kazan and Gardiner, 2018). Infected plants display a chocolate brown discoloration that can extend over one to three internodes along the stem (Chakraborty et al., 2006; Cook, 2010). *Fpg* can spread up to 18 cm above the crown in infected durum wheat (*T. turgidum*) stems, with sporadic detection of the pathogen at the top of the peduncle, but not in the head (Knight et al., 2017). When inoculated onto the stem base, *Fpg* infected the heads of greenhouse-grown plants (Mudge et al., 2006). However, other studies have failed to detect FCR pathogens in the rachis or grains of infected plants with crown rot symptoms under greenhouse conditions (Covarelli et al., 2012; Winter et al., 2013; Moretti et al., 2014; Beccari et al., 2018). Prematurely senescent culms showed greater visual discoloration and contained greater *Fpg* biomass than nonsenescent culms in tetraploid durum wheat and hexaploid spring wheat at the early milk stage (Knight et al., 2017; 2021). Diseased plants exhibit both necrotic and symptomless tissues. Therefore, the transmission patterns of *Fpg* in symptomless tissues of diseased wheat plants with differing levels of FCR severity require further characterization.

In infected wheat, the three FCR pathogens (*Fpg*, *F. culmorum*, and *F. graminearum*) biosynthesize mycotoxins, including deoxynivalenol (DON), zearalenone (ZEN), and DON derivatives (Lemmens et al., 2005; Clear et al., 2006; Winter et al., 2013; Moretti et al., 2014). DON may play a role in stem colonization by *Fpg* and *F. graminearum* (Mudge et al., 2006). Toxins accumulate not only in the stem, but also in the grains of wheat plants showing FCR symptoms caused by the three FCR pathogens (Moretti et al., 2014; Beccari et al., 2018). However, DON and DON-3-glucoside (D3G) were detected in the rachis and husk tissues of wheat plants with FCR caused by *F. culmorum*, but not in the grain, suggesting that systemically translocated toxins might be prevented from entering the grain due to xylem discontinuity in wheat heads (Winter et al., 2013). Other studies have established that DON accumulates in wheat grains, but to a lesser extent than in the stems (Mudge et al., 2006; Moretti et al., 2014; Beccari et al., 2018). The levels of mycotoxins in tissues with FCR symptoms varied greatly among studies, possibly due to differences in the pathogen strains and cultivars examined (Winter et al., 2013; Moretti et al., 2014). However, the distribution of toxins associated with *Fpg* in wheat plants exhibiting different levels of FCR symptoms has not been well studied. Moreover, the transmission of the associated toxins from symptomatic internodes to asymptomatic internodes/head tissues (including apparently healthy tissues) is unclear.

In the present study, we analyzed the distribution of *Fpg* and the accumulation of its associated toxins DON, D3G, 3-acetyldeoxynivalenol (3ADON), 15-acetyldeoxynivalenol (15ADON), nivalenol (NIV), and ZEN, in the internodes and head tissues of inoculated field-grown wheat plants exhibiting differing levels of FCR severity at harvest. Our goal was to examine whether and how the distribution and transmission of *Fpg* and its toxins in wheat plants with FCR are related to disease severity, wheat cultivar, and the distance between asymptomatic tissues and internodes showing visual FCR symptoms. Our findings highlight the utility of visually examining for FCR symptoms in parallel with biomass and toxin accumulation measurements and shed light on pathogen and toxin transmission in asymptomatic tissues of diseased plants.

## 2 Materials and methods

### 2.1 Inoculum source

*Fpg* strain G14LY24-2 was grown on sterilized wheat grains. G14LY24-2, previously obtained from wheat crowns with FCR, was characterized as *Fpg* based on morphological characteristics and the partial sequences of the gene encoding translation elongation factor-1α (GenBank accession no. KX702418.1) (Xu et al., 2018). The inoculum was prepared as follows: After being soaked in water for 12 h, wheat grains (approximately 250–500 g) were placed into polyethylene plastic bags (36 cm × 10 cm × 8 cm), sterilized at 121°C for 1 h, stored in the bags for 2 days, and autoclaved again at 121°C for 1 h. Sterilized grains were spread onto sterile stainless-steel plates (48 cm × 35 cm × 7 cm) to a depth of 2 cm. Each plate was inoculated with mycelium blocks (1 cm × 1 cm) from two 9-cm plates filled with potato dextrose agar (PDA) medium (200 g fresh potato, 20 g glucose, 15 g agar, 1 L distilled water) (Fang, 1998) that had been inoculated with *Fpg* G14LY24-2 and incubated at 25°C for 3 days in the dark. After 2 weeks of incubation at 25°C in the dark, the entire wheat grain surface was colonized by mycelium. The wheat grains were then air-dried for one week to produce the inoculum for treating whole seeds as described below (approximately 100 seeds per 9 g of grains).

### 2.2 Experimental sites and inoculation

FCR data were obtained from experimental wheat fields near Jiaozuo City (Wenxian County; 35°2′30″N, 113°5′40″E, altitude 109.7 m) in 2019 and Anyang City (Neihuang County; 36°6′14″N, 114°54′26″E, altitude 41.0 m), China, in 2020. In these areas, FCR caused by *Fpg* had occurred over three consecutive years. Due to their differing resistance to FCR, two winter wheat cultivars, ‘Bainong 207’ (susceptible) and ‘Aikang 58’ (highly susceptible), were commonly planted in the NCP (Xu et al., 2021a). For this study, Aikang 58 was planted in Wenxian County in 2019, and Bainong 207 was planted in Neihuang County in 2020. Seeds were sown at a rate of 67 seeds/m at a row width of 20 cm. Healthy seeds and fungal inocula were mixed at a ratio of 1:1 (w/w) and mechanically sown with 2B-6-ZL150 autonomous CNC precision seeder (Henan Liken Agricultural Machinery Co., Ltd.) in four replicated experimental plots (1.5 m × 25 m). There was a 1-meter distance between each plots. Control plots were planted in the same manner with healthy uninoculated wheat seeds. The base fertilizer (N-P-K: 25-13-7, the Experimental Factory of the Soil and Fertilizer Institute of Henan Academy of Agricultural Sciences) was mixed with the soil before planting at 90 g/m^2^, and a top dressing of 15 g/m^2^ urea was applied at the jointing stage (Feeke’s GS 5, Large, 1954).

### 2.3 Assessment of FCR symptoms in experimental wheat fields

**Figure 1:**
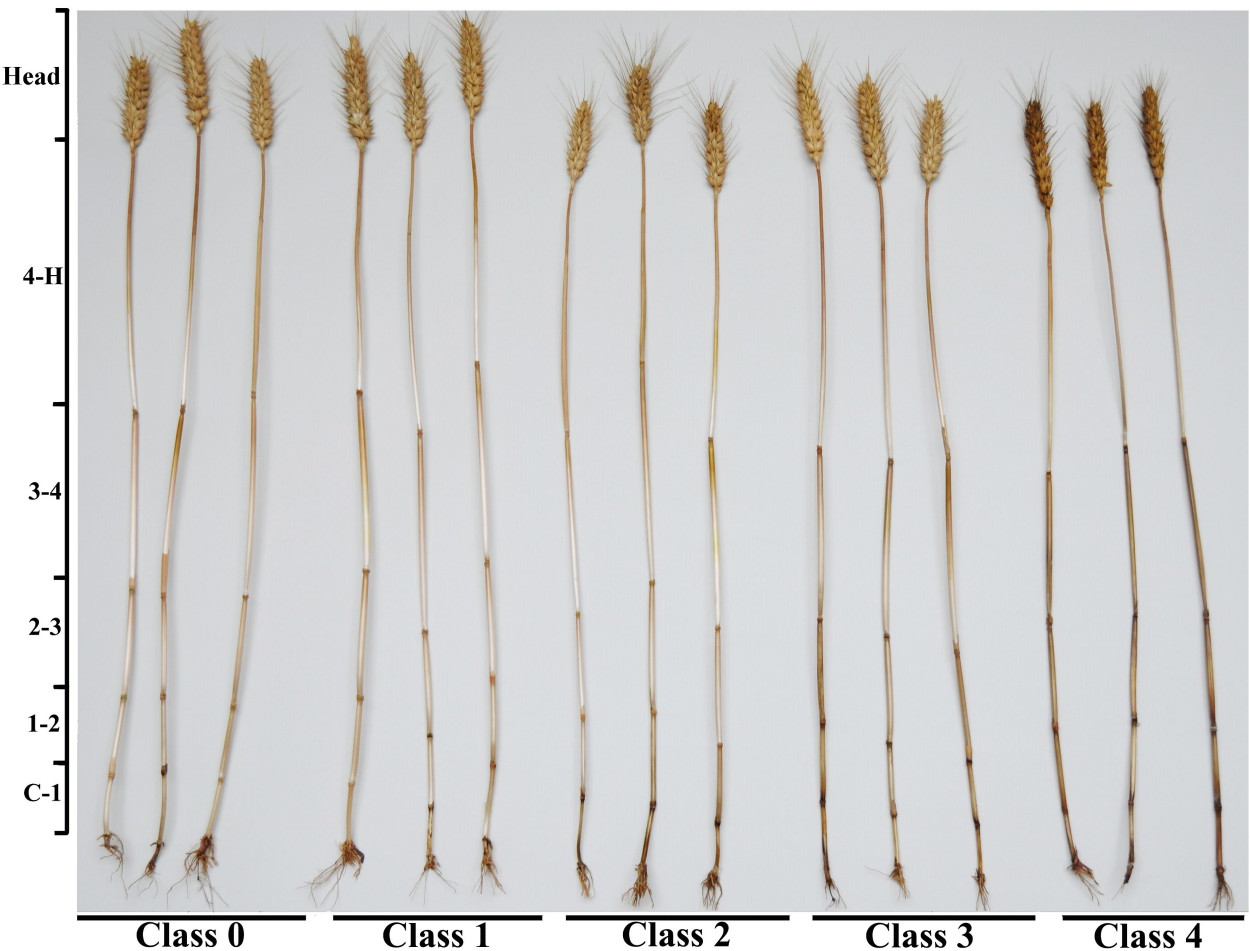
Photographs showing different severity classes of FCR caused by *Fusarium pseudograminearum* in wheat cv. ‘Bainong 207’ grown in Neihuang County, Anyang City, Henan Province, China in 2020. FCR disease severity at the stem base was visually assessed and grouped into five classes, where 0 = no visible lesions, 1 = brown at the point of tiller attachment up to the first internode; 2 = brown up to the second internode; 3 = brown up to the third internode; and 4 = brown up to the fourth internode or above. The stems (with the leaf sheath removed) were divided into eight segments to produce the tissue samples: C-1, first internode; 1-2, second internode; 2-3, third internode; 3-4, fourth internode; 4-H, peduncle; H, head including rachis; husk; and grain.

To avoid the possible influence of other wheat diseases, this work as performed in experimental wheat fields in which only FCR had been observed for three consecutive years. FCR development was monitored at the jointing (Feeke’s GS 5, Large, 1954) and filling stages (Feeke’s GS 11.1, Large, 1954). A total of 120 stems per treatment (30 stems per plot) were collected, washed, and evaluated for the incidence and severity of FCR at the jointing stage. Another 120 stems per treatment (30 stems per plot) were collected, washed, and evaluated for the incidence and severity of FCR at the grain-filling stage based on the following method (Smiley et al., 2005b). FCR severity on the stem was visually assessed and categorized into five classes, where 0 = no visible symptoms and 1, 2, 3, and 4 = brown at the point of tiller attachment up to the first, second, third, and fourth internodes, respectively (**Figure 1**; **Supplementary Figure S1**). The disease severity index (DSI) for each treatment was then calculated as described by Fang (1998): DSI = 100 × [(0 × P_0 +_ 1 × P_1 +_ 2 × P_2 +_ 3 × P_3 +_ 4 × P_4_)]/120, where P_0_, P_1_, P_2_, P_3_, and P_4_ represent the total number of stems with scores of 0, 1, 2, 3, and 4, respectively. The number of white spikes and the total number of spikes were counted in a 0.5-m^2^ area (four sites per plot) in plants at the soft dough stage (Feeke’s GS 11.2, Large, 1954) at 21 days after anthesis. The ratio of the number of white spikes to the total number of spikes was defined as the white spike rate (Fang, 1998). Three small areas (0.5 m^2^) per plot were randomly chosen and harvested using a plot thresher (QKT-320, Weihui Agricultural Machinery Factory of Henan Province) with a fan speed of 1,240 rpm to thresh the spikes in each small plot. Once dried (45°C, 5 h), the spikes were placed in separate mesh bags and stored in room temperature.

### 2.4 Sampling and classification of diseased plants at harvest

For each cultivar, 90 tillers from plants showing each of the five disease severity classes (0 to 4) were collected at harvest and divided into three subgroups. Tillers of plants with disease severity class 0 were collected from control plots, and plants with disease severity classes 1 to 4 were collected from inoculated plots. Each subgroup (with the leaf sheath removed) was further divided into eight segments: C-1, first internode; 1-2, second internode; 2-3, third internode; 3-4, fourth internode; 4-H, peduncle; rachis; husk; and grain. Due to the shriveled kernels resulting from diseased wheat plants, which are light in weight, manual threshing was required during the separation progress. This step yielded a total of 120 samples per cultivar (5 disease classes × 3 subsamples × 8 tissue types).

### 2.5 Pathogen isolation, DNA extraction, and species identification

To isolate *Fpg*, nine individual samples were randomly selected from each tissue type (except husks and grains). The husks and grains were isolated together as a spikelet before manual threshing. This step gave a final total of 675 stem samples (9 individual samples × 5 stem segments× 3 subsamples × 5 severity classes), 135 rachis samples (9 × 1 × 3 × 5), and 135 spikelets (9 × 1 × 3 × 5, including husks and grains) per cultivar. The 675 internode tissues, 135 rachis samples, and 135 spikelets were surface sterilized using 70% (v/v) ethanol for 30 s followed by 3% (w/v) NaClO for 90 s, rinsed three times in sterile distilled water, and plated on PDA medium. Each isolate was picked with a sterile toothpick, cultured on a new PDA plate, and purified by hyphal tipping (Fang, 1998).

DNA was extracted from fungal hyphae using an E.Z.N.A. HP Fungal DNA Kit (Omega Bio-tek, Inc.) following the manufacturer’s protocols for fresh/frozen specimens, and was subsequently analyzed by triplex PCR using the species-specific primers Fp1-1/Fp1-2, FaF/FaR, and FgF/FgR (Aoki and O’Donnell, 1999; Yin et al., 2009; **Table 1**). One hundred milligrams of fresh fungal hyphae were transferred to a 2.0-mL safe-lock tube (Eppendorf), followed by the immediate addition of 500 µL Buffer CPL from the Fungal DNA Kit (Omega Bio-tek, Inc.) and five 3 mm-diameter stainless steel grinding balls. The mixture was shaken and crushed in a Retsch MM400 grinding mill (Verder Shanghai Instruments and Equipment Co., Ltd.) at 30 Hz for 2 min, and then incubated at 65°C for 15 min, with gentle inversion of the tube twice during the incubation period. Then, 500 µL of chloroform/isoamyl alcohol (24:1, v/v) was added and vortexed to ensure thorough mixing.

**Table 1:** Primer pairs used for qualitative and quantitative detection of *Fusarium pseudograminearum*, *F. graminearum*, *F. asiaticum*, and *Triticum* sp. DNA in this study.

Primer	Sequence (5′→3′)	Target	Fragment	Reference
TEF1α.2F	ATCATTCGAATCGCTCGACG			
TEF1α.2R	AAAAATTACGACAAAGCCGTAAAAA	*F. pseudograminearum*	82 bp	Knight et al., 2012
Hor1 F	TCTCTGGGTTTGAGGGTGAC			
Hor2 R	GGCCCTTGTACCAGTCAAGGT	Wheat	63 bp	Nicolaisen et al., 2009
Fp1-1	CGGGGTAGTTTCACATTTCCG			
Fp1-2	GAGAATGTGATGACGACAATA	*F. pseudograminearum*	523 bp	Aoki and O’Donnell, 1999
FaF	CAGCTTCCTCGAAGACCT			
FaR	GGACCGTAAATTTCTTCAGTG	*F. asiaticum*	292 bp	Yin et al., 2009
FgF	TATCCCTTATGGGTCTTGGT			
FgR	GGACCGTAAACTTCTTCTGCA	*F. graminearum*	352 bp	Yin et al., 2009

The sample was then centrifuged at 10,000 g for 5 min and 300 µL of the supernatant was carefully transferred to a new 1.5-mL microfuge tube. The binding conditions were adjusted by adding 150 µL of Buffer CXD, followed by 300 µL of absolute ethanol, and the mixture was vortexed to achieve homogeneity. The entire sample was loaded onto a HiBind DNA column in a 2.0-mL collection tube, which was then centrifuged at 10,000 g. The 2.0-mL collection tube and the flow-through liquid were discarded. The column was transferred to a new collection tube and washed by adding 650 µL of SPW Wash Buffer diluted with 96%–100% ethanol. The column was centrifuged at 10,000 g for 1 min, the flow-through liquid was discarded, and the wash step was repeated. The column was placed back into the collection tube and centrifuged at 10,000 g for 2 min to dry. The column was transferred to a fresh 1.5-mL tube, and 50 µL of sterile deionized water pre-warmed to 65°C was applied. The sample was incubated at room temperature for 2 min and then centrifuged at 10,000 g for 1 min to elute the DNA.

Triplex PCR was carried out in a C1000 Touch Thermal Cycler (Bio-Rad) using the following program: 2 min at 95°C, 40 cycles of (1 min at 95°C, 30 s at 55°C, 90 s at 72°C), followed by 10 min at 72°C. The total reaction volume was 25 µL: 12.5 μL of premix Taq (Takara Taq version 2.0) (Takara Biotechnology Co., Ltd, Dalian, China), 1 μL of template DNA (100 ng/µL), 1 μL of each specific primer for a total of 6 μL of primers (**Table 1**) (10 mM each), and 5.5 μL of RNase-free H_2_O. A negative control (no DNA template) and three positive controls (DNA extracted from *F. pseudograminearum* G14LY24-2, *F. graminearum* G13XX1-4, or *F. asiaticum* mycelia G14XY4-32-1) were included along with each PCR amplification (Xu et al., 2018). G13XX1-4 and G14XY4-32-1, previously obtained from wheat crowns with FCR, was characterized as *F. graminearum* and *F. asiaticum* based on morphological characteristics and the partial sequences of the gene encoding translation elongation factor-1α, respectively (GenBank accession no. KX663782.1 and KX702562.1) (Xu et al., 2018). The PCR products were diluted 2-fold with ddH_2_O for analysis via capillary electrophoresis, which was performed using a LabChip GX Touch HT Automated Bioanalysis System (PerkinElmer) and a DNA 1K Kit (PerkinElmer).

### 2.6 Development of a qPCR assay for *F. pseudograminearum* in different plant tissues

To create a standard curve for DNA quantification from samples, genomic DNA was extracted from pure *Fpg* cultures and healthy wheat grains. After *Fpg* G14LY24-2 was grown on PDA medium for 5 days, mycelia were scraped from the plate with a sterilized toothpick and frozen in liquid nitrogen. Healthy grains of winter wheat cv. Bainong 207 were finely ground in a Retsch ZM200 grinding mill (Verder Shanghai Instruments and Equipment Co., Ltd.) with a 0.5-mm stainless steel ring sieve and passed through a 20-mesh screen. Wheat tissues were stored in 4 °C in the refrigerator. DNA was extracted from wheat tissues using a Sangon Biotech-Ezup Plant Genomic DNA Extraction Kit (Sangon Biotech [Shanghai] Co., Ltd.) as follows: 100 mg of ground wheat tissue was transferred to a 2.0-mL Safe-lock tube (Eppendorf), followed by the addition of 600 µL PCB extraction buffer from the kit and five 3-mm-diameter stainless steel grinding balls. The mixtures were then shaken and crushed in a Retsch MM400 grinding mill (Verder Shanghai Instruments and Equipment Co., Ltd.) at 30 Hz for 2 min, and then incubated at 65°C for 25 min, with occasional mixing.

After 600 µL of chloroform was added to the mixture, samples were mixed well and centrifuged at 10,000 g for 5 min. Then, 300 µL of the supernatant was transferred to a new 1.5-mL microfuge tube. An equal volume of buffer BD was added to the supernatant, mixed by inverting 3 to 5 times, and then an amount of absolute ethanol equal to the supernatant volume was added, fully mixed, applied onto the Sangon DNA column with a pipette, and allowed to stand at room temperature for 2 min. Centrifugation was performed at 10,000 g for 1 min and the flow-through was discarded. After the column was placed back into the collection tube, 500 µL of PW Solution was added, the tube was centrifuged at 10,000 g for 1 min, and the flow-through in the collection tube was removed. Then, 500 µL of Wash Solution was added, the tube was centrifuged at 10,000 g for 1 min, the flow-through was discarded, and the tube was centrifuged at 10,000 g for 2 min to dry. The column was removed and placed into a fresh 1.5-mL microfuge tube. For elution, 50 µL of sterile deionized water was added into the center of the membrane in the column and allowed to stand for 3 min. The tubes were centrifuged at 10,000 g for 2 min to elute the DNA.

Ten-fold serial dilutions of the wheat and fungal genomic DNA standards were initially tested in triplicate to establish a standard curve and to assess PCR efficiencies. The DNA concentrations of the wheat and fungal standards were determined by measuring absorbance at 260 nm using an ultraviolet-visible spectrophotometer [Eppendorf BioSpectrometer Basic, 230 V/50 – 60 Hz (CN)]. Standards were diluted into six 10-fold serial dilutions for fungal DNA (1 pg to 100 ng) and five 10-fold serial dilutions for wheat DNA (100 pg to 1 μg). Quantitative PCR (qPCR) assays were conducted in a CFX96 Touch Real-Time PCR (Bio-Rad) detection system. The qPCR mixtures had a total reaction volume of 20 µL, consisting of 10 µL of 2 × TB Green Premix Ex Taq II (Tli RNaseH Plus) (TaKaRa Biotechnology Co., Ltd., Dalian, China), 2 µL of sample DNA, 1 µL of each primer (4 mM) (**Table 1**), and 6 µL of sterile DNase-free water. The PCR program consisted of 95°C for 3 min, followed by 40 cycles of 95°C for 20 s, 57°C for 25 s, 72°C for 35 s, and 80°C for 3 s. For the wheat DNA assays, qPCR was performed using the following PCR program: 95°C for 3 min; 40 cycles of 95°C for 10 s, 59°C for 10 s, and 72°C for 30 s. PCR standard curves were generated for *Fpg* and wheat DNA via linear regression analysis of known DNA quantities against the corresponding cycle threshold (Ct) values using BIO-RAD CFX Maestro software. For each standard curve, the slope and *y*-intercept of the linear equation (y = mx + q) were estimated, as well as the R^2^ value and reaction efficiency (10^(–1/m)^). The limit of detection of *Fpg* DNA and wheat DNA was 1 pg and 100 pg, respectively.

The linear regression correlations between the Ct values and known quantities of the DNA standards were high for both *Fpg* (y_fungal_ = −3.195x+39.620, R^2^ = 0.984, efficiency [E] = 105.6%) (**Supplementary Figure S2**) and wheat (y_wheat_ = −3.288x + 41.397, R^2^ = 0.995, E = 101.4%) (**Supplementary Figure S3**) DNA. The methods for normalization of the amount of *Fpg* DNA per sample dry weight (Fpg DNA mg/g) and the relative amounts of *Fpg* DNA and wheat DNA (Fpg DNA pg/wheat DNA ng) achieved similar results when comparing the effect of disease class. Extraction of DNA from wheat grain, stem, rachis, and husk tissues provided 334.6–1147.4 ng/mg, 8.1–852.3 ng/mg, 13.5–865.5 ng/mg, and 47.5–1355.7 ng/mg, respectively. Due to discrepancies in the amounts of wheat DNA extracted from the four types of wheat tissues, *Fpg* DNA biomass was reported as the amount of *Fpg* DNA per dry weight of the extracted sample.

### 2.7 Quantification of *F. pseudograminearum* biomass in diseased plants by qPCR

After removing the isolated parts, the remaining parts of each tissue samples (stem, rachis, husk, and grain) were chopped, crushed, and ground with a Retsch ZM200 grinding mill (Verder Shanghai Instruments and Equipment Co., Ltd.) for DNA extraction. To minimize potential contamination between samples, the order of sample grinding in the mill was from low to high disease severity class and from the top to the bottom of the plant. The total weights of the ground samples ranged from 1.17 g to 6.46 g for internode and rachis samples, and were greater than 5 g for husk and grain samples. DNA was extracted from each sample as described above for healthy wheat grains.

Species-specific primer pairs (TEF1α.2F/TEF1α.2R) (Knight et al., 2012) and primers to amplify wheat *translation elongation factor 1α* (Hor1 F/Hor2 R) (Nicolaisen et al., 2009) were used to amplify *Fpg* and wheat DNA, respectively (**Table 1**). To avoid potential interference from FCR caused by *F. graminearum* and *F. asiaticum* from the soil, each DNA sample was analyzed by triplex PCR using the species-specific primer pairs Fp1-1/Fp1-2, FaF/FaR, and FgF/FgR (**Table 1**) and capillary electrophoresis using a LabChip GX Touch HT Automated Bioanalysis System (PerkinElmer) and a DNA 1K Kit (PerkinElmer) as described above. The qPCR assays for *Fpg* and wheat DNA were conducted as described above. A negative control (no DNA template), a positive control (*Fpg* DNA), and another negative control (wheat DNA) were included in parallel with each PCR amplification, using three technical replicates per tissue sample. Normalization of qPCR results was performed using two methods (Knight et al., 2012): analysis of the amount of *Fpg* DNA/wheat DNA and the amount of *Fpg* (ng DNA/mg dry weight of wheat tissue [in mg]). A maximum of 38 cycles (Ct) was determined to be sufficient for generating standard curves and was used as the cut-off to discriminate between positive and negative samples, allowing for accurate qPCR detection.

### 2.8 Analysis of DON, ZEN, and DON derivatives in diseased plants

DON, ZEN, D3G, 3ADON, 15ADON, and NIV contents were quantified in the stem, rachis, husk, and grain samples using 1 g of stem or rachis tissue or 5 g of husk or grain tissue. Toxins were also quantified in grain harvested from an area of 1 m^2^ in each inoculated and uninoculated plot. For grain samples, 20 mL of 50% (v/v) acetonitrile containing 1% (v/v) formic acid was added to a centrifuge tube containing the powdered sample, the sample was vortexed for 5 min and then centrifuged at 5000 g for 5 min. The supernatant (4 mL) was transferred to a 15-mL centrifuge tube containing purification reagents (0.4 g MgSO_4_, 0.2 g NaCl, 0.2 g sodium citrate, and 0.2 g C18 adsorbent), vortexed for 1 min, and centrifuged at 6000 g for 5 min. A 1-mL aliquot of the supernatant was transferred to a fresh tube, evaporated under a nitrogen stream until nearly dry, diluted with 1 mL 50% (v/v) acetonitrile containing 0.1% (v/v) formic acid, passed through a 0.22-μm microporous membrane, and transferred to a chromatographic injection vial for analysis.

Chromatographic separation was performed on an UltiMate 3000 HPLC system (Thermo Fisher Scientific, USA) using a Hypersil Gold C18 reversed-phase chromatographic column (100 mm × 2.1 mm, 1.9 μm, Thermo Fisher Scientific, USA). The mobile phase was composed of methanol and 5 mM ammonium acetate in water. Gradient elution mode was selected, with a flow rate of 0.2 mL/min. After separation, the eluents were analyzed using a Q Exactive-Orbitrap electrostatic orbital trap high-resolution mass spectrometer (Thermo Scientific, USA) in negative ion mode. All toxin standards were purchased from Pribolab (Qingdao, China) and diluted in acetonitrile (100 µg/mL) for use as stock solutions. The detection limits in wheat were 10 µg/kg for DON, NIV, and 3ADON, 3 µg/kg for 15ADON, and 1 µg/kg for D3G. Quantifications were performed using an Ultimate 3000 ultrahigh performance liquid chromatography system coupled with a Q Exactive-Orbitrap High Resolution Mass Spectrometer (Thermo Fisher Scientific, USA). Detailed characteristics of toxin detection are shown in **Table 2**.

**Table 2:** The parameters of quantitative analysis using in an Ultimate 3000 ultrahigh-performance liquid chromatography system coupled with a Q Exactive-Orbitrap High Resolution Mass Spectrometer (LC–MS/MS analysis) for DON, ZEN, NIV, 15ADON, 3ADON, and D3G contents.

Mycotoxins^x^	Molecular ion	Precursor ion (m/z)	RT^z^ (min)	Ion pair^y^ (m/z)	Collision energy (eV)
DON	[DON-H]^-^	295.1171	3.19	265.1070*/247.0961	15
ZEN	[ZEN-H]^-^	317.1380	7.90	175.0380*/273.1481	45
NIV	[NIV+CH_3_COO]^-^	371.1331	2.13	281.1019*/311.1125	15
15ADON	[15ADON+CH_3_COO]^-^	397.1485	5.88	337.1280*/150.0323	10
3ADON	[3ADON-H]^-^	337.1279	5.87	307.1170*/173.0590	15
D3G	[D_3_G+CH_3_COO]^-^	517.1895	3.01	427.1582*/457.1689	25

^x^DON, deoxynivalenol, its derivatives (D3G, DON-3-glucoside; 3ADON, 3-acetyldeoxynivalenol; 15ADON, 15-acetyldeoxynivalenol; and NIV, nivalenol); and ZEN, zearalenone. ^y^ *Quantitative ion. The detection limits of toxins in wheat were 10 µg/kg for DON and NIV, 10 µg/kg for 3ADON, 3 µg/kg for 15ADON, and 1 µg/kg for D3G, respectively. ^z^RT = the retention time of chromatographic peaks.

### 2.9 Data analysis

Because each cultivar was grown at a different site, the isolation frequency of *Fpg*, the *Fpg* biomass, and the DON, D3G, ZEN, 3ADON, 15ADON, and NIV contents were analyzed separately for the two cultivars. Correlations among variables were analyzed using the psych package (Revelle, 2020) in R (R Core Team, 2017). The *Fpg* DNA, DON, and D3G concentrations were log10-transformed with the formula log (x + 1) prior to analysis of variance (ANOVA) to obtain a suitable distribution of the residuals. Based on the distribution characteristics of the data, multiple linear regression analysis was used to establish the association of DON, D3G, and *Fpg* DNA with the samples, disease severity classes, and cultivars. The contribution of each factor to the variability in the isolation frequency of *Fpg*, the amount of *Fpg* DNA, and the DON and D3G contents, were determined based on the ratio of the sum square of the factor to the total sum square in the ANOVA model.

Generalized linear modeling was then used to establish the association of the isolation frequency of *Fpg* with disease severity classes, tissue types, and the cultivars used. Residual errors were assumed to follow a binomial distribution. The effect of disease severity class tissue type, and cultivar were determined using F-test analysis of the equality of two variances (Xu et al., 2021b). The percentage of decrease in the isolation frequency of *Fpg*, *Fpg* biomass, and DON and D3G contents was defined as the ratio of the factor in symptomless tissues to those in the nearest internode of a diseased stem with FCR symptoms. The ratios were calculated with the formula (Factor of the symptomless internodes or head tissues/Factor of the nearest internode with FCR symptoms × 100) in plants of the same disease severity. The Factor included isolation frequency, *Fpg* biomass, and DON and D3G contents. The distance factor was defined as the distance between the asymptomatic tissues and the highest internode with symptoms. Each forward position (C-1, first internode; 1-2, second internode; 2-3, third internode; 3-4, fourth internode; 4-H, peduncle; rachis; husk; and grain) counted as 1. For example, the distance between the peduncle and the first internode in the tillers of a plant with a disease severity class of 1 is 4. The percentage decrease in the isolation frequency of *Fpg*, *Fpg* biomass, and DON and D3G concentrations were log10-transformed with the formula log(x + 1) as described above.

Multiple factor analysis was also used to analyze the correlations between the percentages of decrease in the isolation frequency, biomass, and DON and D3G contents and the disease severity class, distance, or cultivar. Finally, the relationships between the percentages of decrease in the isolation frequency of *Fpg* and *Fpg* DNA, DON, and D3G contents in asymptomatic tissues and the distance were assessed via linear regression. All data analyses were performed in R (v.4.0.0; R Foundation for Statistical Computing, Vienna, Austria).

## 3 Results

### 3.1 *Fusarium pseudograminearum* as the main pathogen causing FCR in the field

Under inoculated field conditions, for wheat cv. Aikang 58 (highly susceptible) grown in Wenxian County in 2019 and cv. Bainong 207 (susceptible) grown in Neihuang County in 2020, the incidence of stems with FCR was 4.4% and 70.5% at the jointing stage (Feekes 5) and 50.4% and 68.6% at the filling stage (Feekes 11.1), respectively. For these plants, the disease index at the filling stage was 31.7 and 36.0, and the percentage of whiteheads was 6.9% and 4.2%, respectively (**Supplementary Table S1**). Of the 102 and 167 *Fusarium* isolates from wheat cv. Aikang 58 and cv. Bainong 207 samples, 97% and 100% of them (99 and 167 isolates, respectively) were *Fpg*, based on analysis using specific PCR primers and morphological characteristics. The other few isolates were *F. graminearum*. *Fusarium* species were absent in the wheat head samples from both cultivars. In this experiments of this study, *Fpg* was the main pathogen causing FCR in the field.

### 3.2 Distribution of *F. pseudograminearum* and toxin accumulation in diseased plants

**Figure 2:**
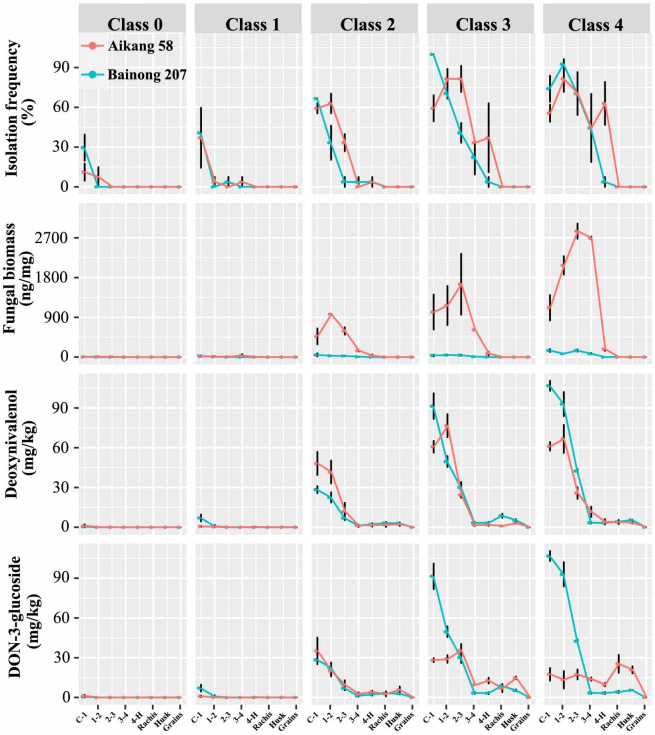
The isolation frequency of *Fusarium pseudograminearum*, *Fusarium pseudograminearum* biomass (Fpg DNA ng/mg), and DON (mg/kg), D3G (mg/kg), ZEN (mg/kg), 3ADON (mg/kg), 15ADON (mg/kg), and NIV (mg/kg) contents in different tissues of wheat with different severity classes of Fusarium crown rot: wheat cv. ‘Aikang 58’ (red) in Wenxian County, Jiaozuo City, Henan Province in 2019 and wheat cv. ‘Bainong 207’ (blue) in Neihuang County, Anyang City, Henan Province in 2020.

For both cultivars, the isolation frequency, *Fpg* biomass, and toxin concentrations decreased with increasing distance from the stem base (first internode), to the grain. The mean results in plants of all disease classes for both cultivars were as follows: *Fpg* isolation frequency went from 53.3% (stem base) to 0.0% (grain); *Fpg* biomass dropped from 531.8 to 0.0 Fpg DNA ng/mg; DON contents decreased from 40.58 to 0.01 mg/kg, D3G contents from 35.04 to 0.05 mg/kg, 15ADON contents from 8.03 to 0.00 mg/kg, 3ADON contents from 7.68 to 0.00 mg/kg, and ZEN contents from 26.87 to 0.00 mg/kg. *Fpg* can be isolated from peduncle tissue of diseased plants classified as class 2 or above in both cultivars (3.7% to 63.0%), but not from head tissues. Moreover, we detected *Fpg* biomass by qPCR in peduncles (0.01 to 180.45 Fpg DNA ng/mg), as well as in rachis and husk tissues (0.01 to 0.70 Fpg DNA ng/mg) (**Figure 2**; **Supplementary Tables S2**, **S3**), but not in grains. The mean DON and D3G concentrations for rachis, husk, and grain tissue were 2.28 and 13.76 mg/kg, 2.29 and 9.69 mg/kg, and 0.01 and 0.05 mg/kg, respectively (**Supplementary Tables S2**, **S3**).

There was a positive (*P* < 0.001) correlation among *Fpg* isolation frequency, *Fpg* biomass, and DON and D3G contents (**Table 3**). Only *Fpg* biomass and D3G contents differed between the two cultivars (*P* < 0.001), whereas the *Fpg* isolation frequency and DON contents did not (**Table 4**). The *Fpg* isolation frequency, *Fpg* biomass, and toxin levels also differed depending on the tissue type and disease severity class (*P* < 0.01) (**Table 4**). The isolation frequency, *Fpg* biomass, and DON and D3G contents at any given internode were significantly (*P* < 0.05) higher for tillers in high FCR disease classes (3 to 4) than for those in low FCR disease classes (0 to 1) (**Figure 2**; **Supplementary Tables S2**, **S3**). The distribution of *Fpg* and toxin accumulation in diseased plants were affected by the tissue type and disease severity class.

**Table 3:** Pearson pairwise correlations among the isolation frequency of *Fusarium pseudograminearum*, *F. pseudograminearum* biomass, and toxin contents (DON, D3G, ZEN, 3ADON, and 15ADON) in four internodes, peduncles, and head tissues of plants with different severity classes of Fusarium crown rot in the field in 2019 and 2020.

	Isolation frequency^y^	*Fpg* biomass	DON	D3G	ZEN	3ADON
2019						
*Fpg* biomass	0.78***	–	–	–	–	–
DON^x^	0.81***	0.63***	–	–	–	–
D3G	0.73***	0.56**	0.72***	–	–	–
ZEN	0.25	0.21	0.44*	0.19	–	–
3ADON	0.78***	0.60***	0.98***	0.69***	0.49**	–
15ADON	0.78***	0.60***	0.98***	0.69***	0.47*	0.99***
2020						
*Fpg* biomass	0.79***	–	–	–		–
DON	0.88***	0.76***	–	–	–	–
D3G	0.76***	0.73***	0.63***	–	–	–
ZEN	0.41*	0.59***	0.66***	0.11	–	–
3ADON	0.83***	0.74***	0.97***	0.59***	0.68***	–

^y^Asterisks indicate correlation coefficient significant at P = 0.05 (*), P = 0.01 (**), and P = 0.001 (***).

^x^DON, deoxynivalenol, its derivatives (D3G, DON-3-glucoside; 3ADON, 3-acetyldeoxynivalenol; 15ADON, 15-acetyldeoxynivalenol; and NIV, nivalenol); and ZEN, zearalenone.

**Table 4:** Effects of disease severity class, tissue type, and cultivar on the isolation frequency of *Fusarium pseudograminearum*, *F. pseudograminearum* biomass, and DON and D3G contents.

Factors	Isolation frequency			*F. pesudograminearum* DNA content			DON content^x^			D3G content		
	Deviance	F	P	Sum Sq	F	P	Sum Sq	F	P	Sum Sq	F	P
Disease severity classes	15.11	3.78	0.004	18.85	14.04	<0.001	14.39	38.57	<0.001	22.33	62.81	<0.001
Segment samples	22.86	3.27	0.002	47.81	20.34	<0.001	12.37	18.94	<0.001	8.90	14.30	<0.001
Cultivar	0.23	0.23	0.630	8.95	26.64	<0.001	0.03	0.34	0.561	3.07	34.59	<0.001
Residuals	43.85			22.49			6.25			5.95		
Total variation	82.05			98.10			33.04			40.25		

^x^DON, deoxynivalenol; D3G, DON-3-glucoside. Multiple factor analysis of *Fusarium pseudograminearum* biomass, DON, and D3G content on different disease severity classes, segment samples, and cultivars. The *F. pseudograminearum* DNA, DON, and D3G content is depicted as the log(x+1) of the their content. The degree of freedom of cultivar, disease severity classes, segment samples, and residuals are 4, 7, 1, and 67, respectively. Based on the distribution characteristics of data, multiple linear regression analysis was then used to establish the association of DON, D3G, and *F. pseudograminearum* DNA with segment samples, disease severity classes, and cultivar. The contribution of each factor to the variability of the isolation frequency of *F. pseudograminearum*, the *F. pseudograminearum* DNA, DON, and D3G content variation was determined by the ratio of the factor’s sum square to the total sum square in the ANOVA model. Generalized linear modeling (GLM) was then used to establish the association of the isolation frequency of *F. pseudograminearum* with disease severity classes, segment samples, and cultivar used. Residual errors were assued to follow a binomial distribution. The effect of disease severity classes, segment samples, and cultivars were determined based on the F test of deviences.

### 3.3 *F. pseudograminearum* biomass and toxin accumulation in asymptomatic tissues

**Figure 3:**
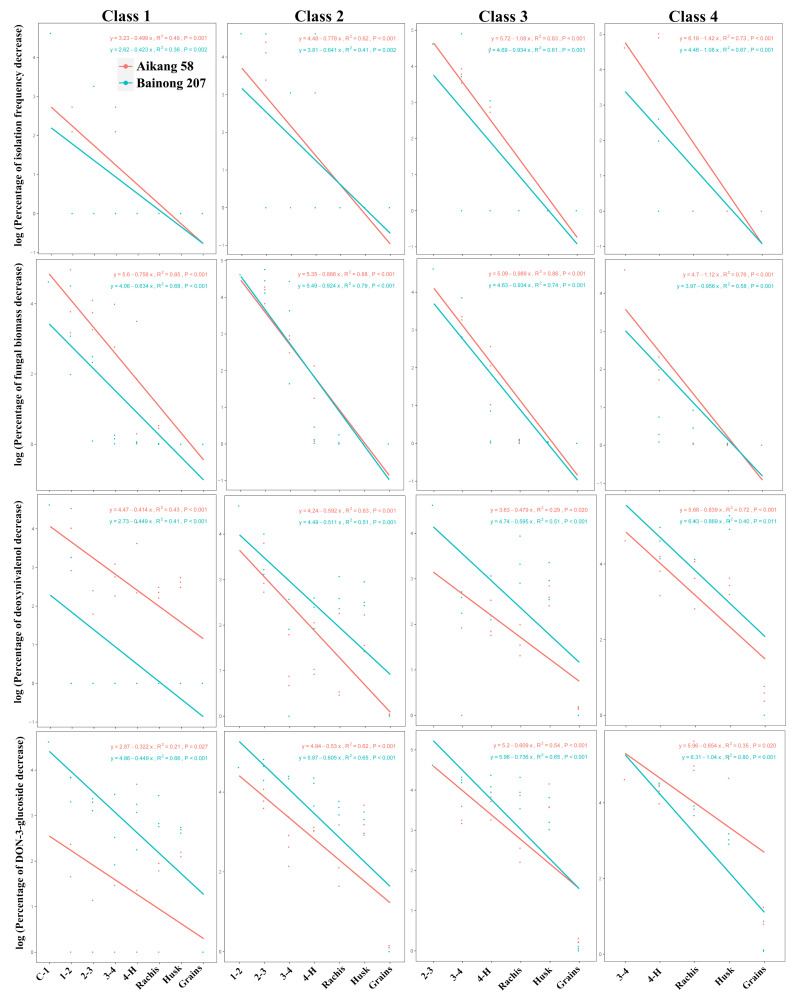
Linear relationship between the logarithm of the percentage of decrease in isolation frequency of *Fusarium pseudograminearum*, *F. pseudograminearum* biomass, DON content, and D3G content per sample (n = 3) vs. distance from the internode with disease symptoms, disease severity classes, and cultivar.

The extent of decrease in the isolation frequency of *Fpg*, *Fpg* biomass, and DON and D3G contents in asymptomatic tissues of diseased plants from both cultivars was related to the distance between asymptomatic tissues and the nearest internode with FCR symptoms (*P* < 0.01) and to the disease severity classes (*P* < 0.001) (**Table 5**, **Figure 3**).

**Table 5:** Multiple factor analysis of the percentage of decrease in isolation frequency of *Fusarium pseudograminearum*, *F. pseudograminearum* biomass, and DON and D3G contents in two wheat cultivars vs. disease severity classes and distance from a diseased internode.

Factors	Percentage of decrease of isolation frequency			Percentage of decrease of *F. pesudograminearum* DNA content			Percentage of decrease of DON content^x^			Percentage of decrease of D3G content		
	Sum Sq	F	P	Sum Sq	F	P	Sum Sq	F	P	Sum Sq	F	P
Cultivar	0.13	2.46	0.120	9.32	11.56	<0.001	0.74	0.47	0.493	8.63	5.96	0.016
Disease severity classes	0.92	17.05	<0.001	6.16	7.64	0.006	55.51	35.40	<0.001	36.74	25.38	<0.001
Distance	18.94	50.37	<0.001	461.64	81.79	<0.001	196.75	17.93	<0.001	163.91	16.17	<0.001
Residuals	7.84			117.72			228.90			211.41		
Total variation	27.83			594.84			481.90			420.69		

^x^DON, deoxynivalenol; D3G, DON-3-glucoside. The isolation frequency, *F. pseudograminearum* DNA, DON, and D3G content is depicted as the log(x+1) of the their content as a percentage of their content of the highest internode with symptom in the diseased plants. The percentage of decrease of isolation frequency of *F. pseudograminearum*, *F. pseudograminearum* biomass, DON, D3G content were defined as the ratio of the symptomless internodes/head tissues to the nearest internode with FCR symptoms of a diseased stem. The ratio were calculated with the formula (Factor of the symptomless internodes or head tissues/Factor of the internode with FCR symptoms ×100)% under the same severity of disease conditions. “Distance” refers to distance between the asymptomatic tissues and highest internode with symptom. The degree of freedom of cultivar, disease severity classes, distance, and residuals are 1, 1, 7, and 146, respectively.

The two cultivars differed in the distribution of *Fpg* (*P* < 0.001) and D3G contents (*P* = 0.016) in asymptomatic tissues of diseased plants, but not in the isolation frequency or DON contents (**Table 5**). Based on the different slopes of the curves, the transmission rates of *Fpg* biomass and the levels of its toxins (DON and D3G) in asymptomatic tissues of diseased plants were related to the disease severity classes and cultivars (**Figure 3**).

## 4 Discussion

In this study, our goal was to test whether the distribution and transmission of *Fpg* and its toxins in winter wheat plants exhibiting FCR symptoms is related to visual disease severity, wheat cultivar, and sample tissue. To this end, we investigated the isolation frequency of *Fpg*, *Fpg* biomass, and accumulation of the associated toxins in a variety of wheat tissues showing differing levels of FCR severity. *Fpg* biomass and toxin content decreased with increasing vertical distance from the stem base, where FCR symptoms are usually most severe. We detected positive (*P* < 0.001) correlations among *Fpg* isolation frequency, *Fpg* biomass, DON content, and D3G content. Strong correlations between fungal biomass and symptom severity in wheat seedlings were previously reported by Knight et al. (2012), suggesting that mycotoxins produced by *Fpg* are associated with disease progression. However, another study indicated that the presence of DON is not required for crown rot disease symptoms (Mudge et al., 2006). In the previous study, the day-time conditions included a temperature of 22°C and relative humidity of 60%, with an 18-hour photoperiod, while the night-time temperature was maintained at 18°C with 80% RH in controlled environmental rooms (Mudge et al., 2006). However, in this study, the samples were grown in a natural environment, which could explain the observed differences.

The isolation frequency of *Fpg*, *Fpg* biomass, and mycotoxin accumulation were also affected by the tissue type or disease severity class. However, the isolation frequency and DON contents were not related to the differential levels of susceptibility presented by the two cultivars used in this study. In a related study with plants showing differing levels of FCR severity, prematurely senescent culms showed greater visual discoloration and contained greater *Fpg* biomass than nonsenescent culms in tetraploid durum wheat and hexaploid spring wheat at the early milk stage (Knight et al., 2017, 2021).

We compared the extent of colonization of culm/head tissues in the two cultivars based on *Fpg* biomass and toxin accumulation, and observed significant differences between these two susceptible genotypes. As the distance between the sampled tissues and the nearest internode showing symptoms increased, the pathogen isolation frequency and DON content in asymptomatic tissues of diseased plants did not significantly differ between the susceptible genotype Bainong 207 and the highly susceptible genotype Aikang 58 (**Table 5**).

Our qPCR analysis showed that the distribution of fungal biomass from the symptomatic internode to asymptomatic tissues of diseased plants did differ between the two cultivars. We also detected the accumulation of fungal biomass in asymptomatic tissues of both cultivars, suggesting that initial internal colonization/infection may not be affected by the resistance of a particular cultivar. However, *Fpg* biomass and D3G content were significantly different between the two cultivars. These differences may not only be due to the different degrees of susceptibility displayed by the two cultivars, but also affected by other factors, such as differences in climate conditions.

Except for head tissue, the isolation frequency and *Fpg* biomass increased with increasing FCR severity and decreased with increasing distance of the sample from the crown. Culm discoloration is an indicator of *Fpg* biomass in plants in the field (Knight and Sutherland, 2015). We found that the higher the sampling site in the plant, the lower the pathogen isolation frequency and the *Fpg* biomass were. We also demonstrated that pathogens could be isolated from plant tissues lacking visual FCR symptoms, in agreement with the results of previous studies with *Fpg* (Mudge et al., 2006; Xu et al., 2016) and the related species *F. graminearum* (Sieber et al., 1988). These findings confirm the observation that, under certain environmental conditions, *Fpg* can colonize the stem without causing macroscopic symptoms (Knight et al., 2017). This phenomenon may also result from saprophytic growth of *Fpg* after host senescence.

*Fpg* could be isolated from the peduncle in symptomatic samples with severity classes of 2 and above. However, *Fpg* biomass ranged from 0.01 to 180.45 ng DNA/mg in the peduncle at maturity for all severity classes. Knight et al. (2017); Knight et al. (2021) detected the occasional presence of *Fpg* DNA in the peduncles of plants with severe FCR during the early milk development stage in durum wheat, but not in hexaploid spring wheat. Sampling time may be another important reason for the difference in our results compared to those in previous studies. In wheat cv. ‘Sunlin’, *Fpg* biomass was greatest in prematurely senescent culms during the soft dough stage, followed by a decrease at crop maturity (Knight et al., 2021).

In the present study, we did not isolate *Fusarium* spp. from the head tissues of plants in any classes of disease severity, but we did detect *Fpg* DNA in the husk and rachis of plants with high disease severity (3 and 4). Previous studies of plants grown in a growth chamber showed that neither *F. graminearum* nor *F. culmorum* were able to colonize head tissues of wheat affected by FCR caused by pathogens (Winter et al., 2013; Moretti et al., 2014). Similar results were obtained in greenhouse studies with *Fpg*, *F. graminearum*, and *F. culmorum* (Beccari et al., 2018). However, other studies showed that stem colonization by these fungi could extend into head tissues under controlled environmental conditions (Mudge et al., 2006). Two possible explanations may account for these different results. First, in the present study, diseased plants were grown in the field for approximately 240 days, whereas in previous studies, plants were generally grown under greenhouse conditions for only about 90 days. Second, the Aikang 58 and Bainong 207 plants grown in China were shorter than those used in the other studies, so that the rachis was closer to the initial site of infection by the pathogens.

In addition to causing severe yield losses (Murray and Brennan, 2009; Obanor and Chakraborty, 2014), FCR of wheat caused by *Fpg* can also result in mycotoxin accumulation in grains (Moretti et al., 2014; Fan et al., 2021), posing a health hazard to humans and livestock. Because DON is glycosylated by plants to form D3G as a form of detoxification, numerous DON derivatives occur along with DON, increasing the risk of toxicity (Berthiller et al., 2005, 2009). In the present study, DON contents were higher in rachis tissue than in husks, which in turn were higher than in grains. By contrast, D3G contents were much higher in the rachis and husk than in grains. These results are consistent with previous findings examining DON (Covarelli et al., 2012; Moretti et al., 2014; Beccari et al., 2018) and D3G (Winter et al., 2013) occurrence in wheat plants. There is a barrier at the interface between the grain and rachilla, described as a xylem discontinuity (Winter et al., 2013), which could limit the movement of DON and its derivatives into the grain. In addition, the present results suggest that toxin accumulation in the grain may be affected by climate, soil type, and other location-specific factors.

In our study, we did not detect *Fpg* in grains by either physical isolation or qPCR, but we did detect DON and D3G in grains collected from selected diseased plants of the same disease class (**Supplementary Table S2**). These results suggest that DON and D3G could be transported from the stem base to the grain, providing a possible explanation for the finding of a previous study that DON appeared in the grains of field-grown plants, even though no pathogen was detected there (Xu et al., 2008; Fan et al., 2021). However, in the present study, we tested grains collected from plots in the field but did not detect any toxins (**Supplementary Table S1**). This result may have been due to the low toxin contents in the grains of diseased plants: hence, the toxin levels in a pooled sample of grains from the diseased plot was not above the detection limit. Although there is little risk from toxins in the grains of plants with FCR, there were high amounts of toxin in the stems. This result highlights the risk of using straw from wheat fields affected by FCR, a practice that can enhance the spread of FCR.

## 5 Conclusions

In this study, we demonstrated that visual assessment of FCR symptoms in the internodes of diseased wheat plants not only reflects the isolation frequency of *Fpg*, *Fpg* biomass, and mycotoxin (DON and D3G) contents, but also provides important information about pathogen distribution and mycotoxin accumulation in asymptomatic tissues of the diseased plants. The distribution of mycotoxins and the transmission rate of pathogens in asymptomatic tissues of diseased plants were both related to the distance from the internode with disease symptom and to the disease severity class. Interestingly, the two cultivars studied here differed in the distribution of *Fpg* biomass in asymptomatic tissues of diseased plants. Further investigation of *Fpg* occurrence, biomass accumulation, and transmission in different susceptible or resistant cultivars grown in the same site will improve our understanding of the interactions between genotype and *Fpg* during FCR disease development, and should lead to effective strategies for FCR prevention.

## Data availability statement

Data is deposited in National Microbiology Data Center (NMDC) with accession numbers NMDCX0000286 (https://nmdc.cn/resource/genomics/attachment/detail/NMDCX0000286).

## Author contributions

FX: Conceptualization, Data curation, Formal analysis, Investigation, Methodology, Project administration, Resources, Writing – original draft, Writing – review & editing. RS: Data curation, Writing – original draft. LL: Investigation, Methodology, Writing – original draft. SL: Data curation, Methodology, Writing – original draft. JW: Investigation, Writing – original draft. ZH: Investigation, Writing – original draft. WL: Software, Writing – original draft. HW: Data curation, Methodology, Writing – original draft. JL: Data curation, Methodology, Writing – original draft. JF: Methodology, Writing – original draft. AW: Data curation, Software, Writing – original draft. CF: Investigation, Methodology, Writing – original draft. YS: Investigation, Methodology, Writing – original draft. YZ: Writing – review & editing, Formal analysis. XX: Data curation, Writing – review & editing.

## Supplementary material

Supplementary Figure 1 Photographs showing different severity classes of FCR caused by *Fusarium pseudograminearum* in wheat cv. ‘Aikang 58’ grown in Wenxian County, Jiaozuo City, Henan Province, China in 2019. FCR disease severity at the stem base was visually assessed and grouped into five classes, where 0 = no visible lesions, 1 = brown at the point of tiller attachment up to the first internode; 2 = brown up to the second internode; 3 = brown up to the third internode; and 4 = brown up to the fourth internode or above. The stems (with the leaf sheath removed) were divided into eight segments to produce the tissue samples: C-1, first internode; 1-2, second internode; 2-3, third internode; 3-4, fourth internode; 4-H, peduncle; H, head including rachis; husk; and grain.

Supplementary Figure 2 The standard curve graphs of the *Fusarium pseudograminearum* DNA.

Supplementary Figure 3 The standard curve graphs of the *Triticum* sp. DNA.
