# Supplementary figures and images for: Fusarium pseudograminearum biomass and toxin accumulation in wheat tissues with and without Fusarium crown rot symptoms
Source: Front Plant Sci. 2024 May 21;15:1356723. doi: 10.3389/fpls.2024.1356723 (PMC11148387; doi:10.3389/fpls.2024.1356723)

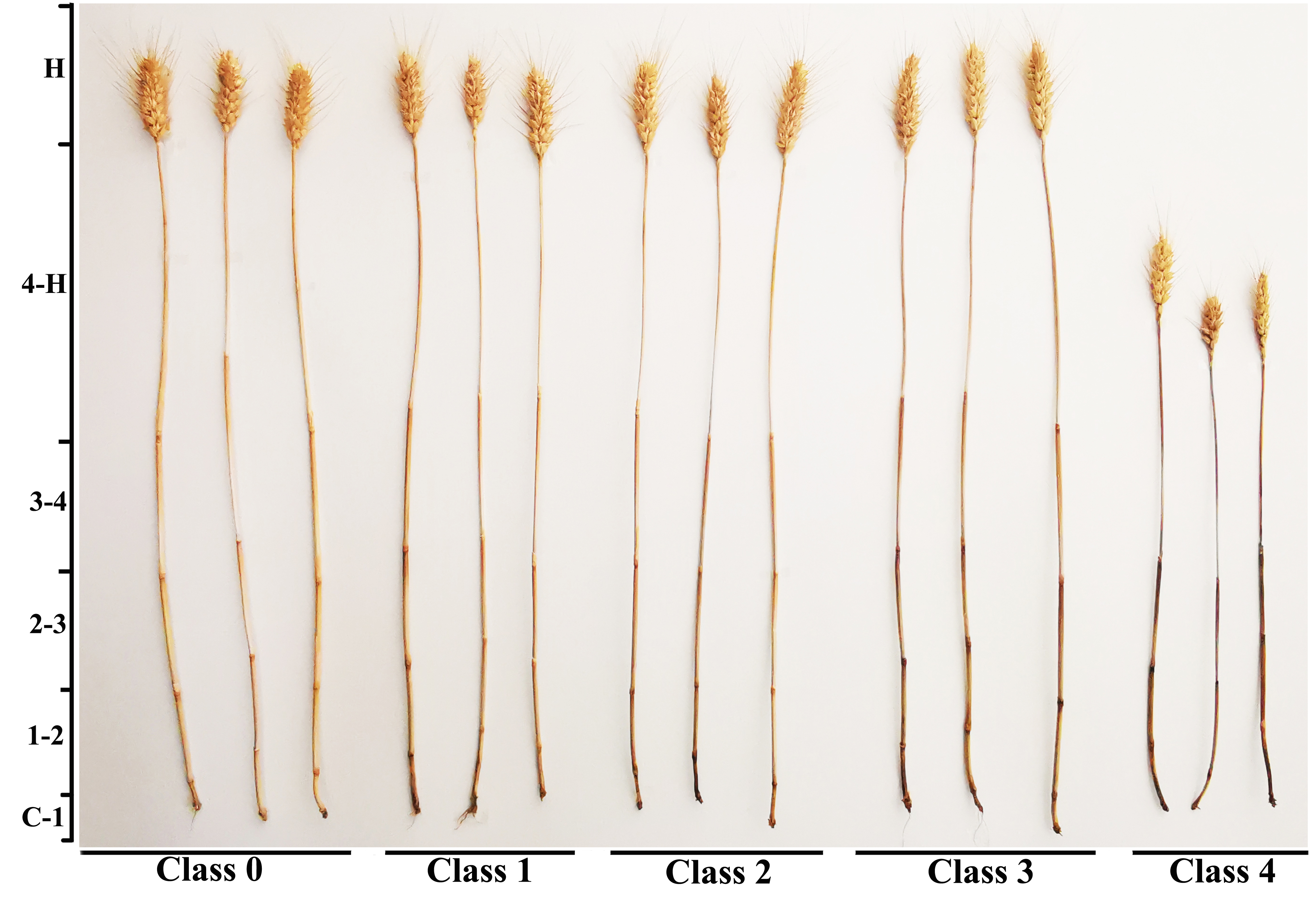

Supplement: Supplementary Figure 1 — Photographs showing different severity classes of FCR caused by Fusarium pseudograminearum in wheat cv. ‘Aikang 58’ grown in Wenxian County, Jiaozuo City, Henan Province, China in 2019. FCR disease severity at the stem base was visually assessed and grouped into five classes, where 0 = no visible lesions, 1 = brown at the point of tiller attachment up to the first internode; 2 = brown up to the second internode; 3 = brown up to the third internode; and 4 = brown up to the fourth internode or above. The stems (with the leaf sheath removed) were divided into eight segments to produce the tissue samples: C-1, first internode; 1-2, second internode; 2-3, third internode; 3-4, fourth internode; 4-H, peduncle; H, head including rachis; husk; and grain. [file Image_1.jpeg]

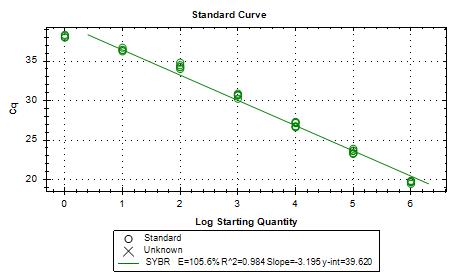

Supplement: Supplementary Figure 2 — The standard curve graphs of the Fusarium pseudograminearum DNA. [file Image_2.jpeg]

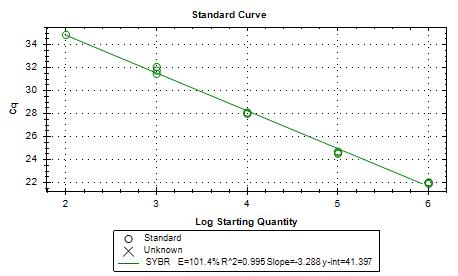

Supplement: Supplementary Figure 3 — The standard curve graphs of the Triticum sp. DNA. [file Image_3.jpeg]
