# Supplementary material for: Fusarium pseudograminearum biomass and toxin accumulation in wheat tissues with and without Fusarium crown rot symptoms
Source: Front Plant Sci. 2024 May 21;15:1356723. doi: 10.3389/fpls.2024.1356723 (PMC11148387; doi:10.3389/fpls.2024.1356723)
Supplement: Supplementary file 4 [file Table_1.docx]

Table S1. Fusarium crown rot severity and toxin content of grain in inoculated and control wheat plots in cultivar cv. ‘Aikang 58’ of Wenxian County in 2019 and cultivar cv. ‘Bainong 207’ of Neihuang County in 2020.

| Assay parameter |  | 2019 Wenxian |  |  | 2020  Neihuang |  |
| --- | --- | --- | --- | --- | --- | --- |
| Year and location |  | Control | Inoc |  | Control | Inoc |
| Disease incidence of rotted stems at Feeke’s GS 5 (%) |  | 0.0±0.0 | 4.4±2.9 |  | 41.2±8.0 | 70.5±13.9 |
| Disease incidence of rotted stems at Feeke’s GS 11.1(%) |  | 28.7±10.8 | 50.4±12.0 |  | 28.6±6.6 | 68.6±5.0 |
| Disease index at Feeke’s GS 11.1 |  | 12.0±4.7 | 31.7±13.9 |  | 12.5±4.6 | 36.0±3.6 |
| Percentage of whiteheads at Feeke’s GS 11.1 (%) |  | 0.6±0.3 | 6.9±1.8 |  | 6.5±1.5 | 4.2±0.3 |
| Toxin content of grain harvested from plots (mg/kg)^y^ |  | 0.0±0.0 | 0.0±0.0 |  | 0.0±0.0 | 0.0±0.0 |

^y^ DON，ZEN，D3G，3ADON, 15ADON and NIV contents were measured in grain. The 0.000±0.000 indicated not dectected. The detection limits of toxins were in wheat 10 µg/kg for DON and NIV, 10 µg/kg for 3ADON, 3 µg/kg for 15ADON, and 1 µg/kg for D3G. The quantitative analysis were performed in an Ultimate 3000 ultrahigh performance liquid chromatography coupled with Q Exactive-Orbitrap High Resolution Mass Spectrometer (Thermo Fisher Scientific, USA). Toxin contents of grain were also quantified in grain harvested from each plots. Plots were harvested with three small areas (0.5 m^2^) per plot, using plot thresher (QKT-320, Weihui Agricultural Machinery Factory of Henan Province) with fan speed of 1,240 rpm to thresh the spikes each small plot. After drying, they are packed in mesh bags separately. “Inoc” refers to plots in which inoculum consisting of wheat grain colonized by *F. pseudograminearum* was banded at a ratio of 1:1 by weight and mixed with wheat seed at the time of planting.
