# Supplementary material for: Fusarium pseudograminearum biomass and toxin accumulation in wheat tissues with and without Fusarium crown rot symptoms
Source: Front Plant Sci. 2024 May 21;15:1356723. doi: 10.3389/fpls.2024.1356723 (PMC11148387; doi:10.3389/fpls.2024.1356723)
Supplement: Supplementary file 5 [file Table_2.docx]

Table S2. The isolation frequency of *Fusarium pseudograminearum*, *Fusarium pseudograminearum* biomass (Fpg DNA ng/mg), DON (mg/kg), D3G content (mg/kg), ZEN (mg/kg), 3ADON (mg/kg), 15ADON (mg/kg), and NIV (mg/kg) in different tissues of cv. 'Aikang 58' in Wenxian County, Jiaozuo City, Henan Province in 2019 under different classes of Fusarium crown rot.

|  |  | Disease severity classes for tillers^y^ | | | | |  |
| --- | --- | --- | --- | --- | --- | --- | --- |
| Characteristic^z^ |  | Class 0 | Class 1 | Class 2 | Class 3 | Class 4 | LSD_0.05_ |
| isolation frequency% |  |  |  |  |  |  |  |
| C-1 |  | 11.1±6.4 b | 37±22.5 ab | 59.3±3.7 a | 59.3±9.8 a | 55.6±6.4 a | 29.7 |
| 1-2 |  | 7.4±7.4 c | 3.7±3.7 c | 63±7.4 b | 81.5±7.4 a | 81.5±9.8 a | 17.3 |
| 2-3 |  | 0.0±0.0 c | 0.0±0.0 c | 33.3±6.4 b | 81.5±9.8 a | 70.4±14.8 a | 25.4 |
| 3-4 |  | 0.0±0.0 b | 3.7±3.7 b | 0.0±0.0 b | 33.3±0 a | 44.4±12.8 a | 14.2 |
| 4-H |  | 0.0±0.0 b | 0.0±0.0 b | 3.7±3.7 b | 37.0±25.9 a | 63.0±16.1 a | 29.4 |
| Rachis |  | 0.0±0.0 a | 0.0±0.0 a | 0.0±0.0 a | 0.0±0.0 a | 0.0±0.0 a | ns |
| Husk |  | 0.0±0.0 a | 0.0±0.0 a | 0.0±0.0 a | 0.0±0.0 a | 0.0±0.0 a | ns |
| Grains |  | 0.0±0.0 a | 0.0±0.0 a | 0.0±0.0 a | 0.0±0.0 a | 0.0±0.0 a | ns |
| DNA (Fpg DNA ng/mg) |  |  |  |  |  |  |  |
| C-1 |  | 6.213±4.951 b | 14.066±5.973 b | 468.772±177.953 a | 1017.887±395.717 a | 1115.742±286.873 a | 0.787 |
| 1-2 |  | 8.940±7.125 b | 11.732±7.250 b | 970.161±8.858 a | 1165.943±442.030 a | 2074.963±205.146 a | 0.801 |
| 2-3 |  | 6.588±6.546 b | 6.972±3.580 b | 590.716±79.852 a | 1648.466±688.784 a | 2850.815±172.11 a | 0.855 |
| 3-4 |  | 0.053±0.041 d | 34.863±30.108 c | 146.675±19.526 b | 614.904±0.101 ab | 2694.509±31.710 a | 0.887 |
| 4-H |  | 0.107±0.097 d | 2.412±2.316 cd | 32.562±21.369 bc | 79.358±42.977 ab | 180.445±36.642 a | 0.862 |
| Rachis |  | 0.000±0.000 b | 0.048±0.040 b | 0.020±0.001 b | 0.179±0.066 b | 0.703±0.041 a | 0.086 |
| Husk |  | 0.000±0.000 b | 0.000±0.000 b | 0.000±0.000 b | 0.124±0.051 a | 0.086±0.054 ab | 0.040 |
| Grains |  | 0.000±0.000 a | 0.000±0.000 a | 0.000±0.000 a | 0.000±0.000 a | 0.000±0.000 a | ns |
| DON (mg/kg)^x^ |  |  |  |  |  |  |  |
| C-1 |  | 1.191±0.945 b | 0.597±0.302 b | 48.154±8.610 a | 60.755±4.368 a | 61.037±3.167 a | 0.301 |
| 1-2 |  | 0.082±0.051 c | 0.345±0.277 c | 41.683±8.611 b | 76.652±8.65 ab | 66.673±10.540 a | 0.213 |
| 2-3 |  | 0.025±0.025 c | 0.033±0.033 c | 11.947±6.487 b | 24.427±2.131 a | 25.869±4.397 a | 0.325 |
| 3-4 |  | 0.000±0.000 b | 0.085±0.049 b | 1.202±0.815 ab | 1.654±0.551 b | 11.631±3.893 a | 0.593 |
| 4-H |  | 0.005±0.005 c | 0.207±0.155 bc | 1.652±1.099 ab | 1.852±0.712 a | 4.173±1.928 a | 0.353 |
| Rachis |  | 0.000±0.000 b | 0.058±0.031 b | 1.766±1.542 ab | 1.005±0.196 ab | 3.980±1.567 a | 0.378 |
| Husk |  | 0.027±0.006 b | 0.083±0.036 b | 2.325±1.180 b | 3.106±0.342 a | 3.461±0.553 a | 0.222 |
| Grains |  | 0.000±0.000 c | 0.000±0.000 c | 0.017±0.006 bc | 0.044±0.008 ab | 0.078±0.031 a | 0.019 |
| D3G (mg/kg) |  |  |  |  |  |  |  |
| C-1 |  | 1.209±0.724 b | 0.887±0.480 b | 35.200±9.967 a | 28.170±1.443 a | 17.484±4.694 a | 0.349 |
| 1-2 |  | 0.029±0.014 b | 0.053±0.027 b | 20.040±4.054 a | 29.032±2.85 a | 13.365±6.528 a | 0.548 |
| 2-3 |  | 0.003±0.003 d | 0.012±0.012 d | 9.534±3.332 c | 35.556±4.708 a | 17.414±3.756 b | 0.243 |
| 3-4 |  | 0.000±0.000 c | 0.019±0.019 c | 2.704±1.116 b | 9.461±0.058 a | 13.881±1.138 a | 0.183 |
| 4-H |  | 0.004±0.004 c | 0.130±0.106 c | 4.109±0.975 b | 12.746±2.164 a | 9.707±1.183 a | 0.168 |
| Rachis |  | 0.000±0.000 c | 0.046±0.025 c | 2.762±1.858 b | 6.820±2.842 b | 25.407±6.750 a | 0.385 |
| Husk |  | 0.023±0.009 c | 0.087±0.018 c | 5.752±2.522 b | 14.750±0.994 a | 20.871±2.625 a | 0.228 |
| Grains |  | 0.000±0.000 c | 0.000±0.000 c | 0.027±0.010 c | 0.095±0.005 b | 0.232±0.054 a | 0.027 |
| ZEN (mg/kg) |  |  |  |  |  |  |  |
| C-1 |  | 0.000±0.000 b | 0.000±0.000 b | 0.026±0.009 b | 0.158±0.046 b | 1.208±0.395 a | 0.123 |
| 1-2 |  | 0.000±0.000 a | 0.000±0.000 a | 0.007±0.007 a | 0.015±0.009 a | 0.003±0.003 a | 0.007 |
| 2-3 |  | 0.000±0.000 b | 0.000±0.000 b | 0.000±0.000 b | 0.005±0.005 b | 0.036±0.021 a | 0.013 |
| 3-4 |  | 0.000±0.000 b | 0.000±0.000 b | 0.000±0.000 b | 0.000±0.000 b | 0.015±0.001 a | 0.001 |
| 4-H |  | 0.000±0.000 a | 0.000±0.000 a | 0.000±0.000 a | 0.000±0.000 a | 0.005±0.005 a | 0.003 |
| Rachis |  | 0.000±0.000 a | 0.000±0.000 a | 0.000±0.000 a | 0.000±0.000 a | 0.000±0.000 a | ns |
| Husk |  | 0.000±0.000 a | 0.000±0.000 a | 0.000±0.000 a | 0.000±0.000 a | 0.000±0.000 a | ns |
| Grains |  | 0.000±0.000 a | 0.000±0.000 a | 0.000±0.000 a | 0.000±0.000 a | 0.000±0.000 a | ns |
| 3ADON (mg/kg) |  |  |  |  |  |  |  |
| C-1 |  | 0.273±0.273 b | 0.050±0.050 b | 2.955±0.836 a | 5.480±1.006 a | 5.737±1.491 a | 0.267 |
| 1-2 |  | 0.030±0.030 c | 0.000±0.000 c | 3.033±0.982 b | 7.681±1.080 a | 4.028±1.116 b | 0.235 |
| 2-3 |  | 0.000±0.000 b | 0.000±0.000 b | 0.838±0.777 b | 2.134±0.536 a | 2.200±0.152 a | 0.261 |
| 3-4 |  | 0.000±0.000 b | 0.000±0.000 b | 0.049±0.049 b | 0.000±0.000 b | 0.981±0.049 a | 0.032 |
| 4-H |  | 0.000±0.000 a | 0.000±0.000 a | 0.018±0.018 a | 0.022±0.022 a | 0.292±0.254 a | 0.114 |
| Rachis |  | 0.000±0.000 a | 0.000±0.000 a | 0.051±0.051 a | 0.000±0.000 a | 0.079±0.056 a | 0.040 |
| Husk |  | 0.000±0.000 b | 0.000±0.000 b | 0.102±0.061 a | 0.064±0.009 ab | 0.056±0.030 ab | 0.038 |
| Grains |  | 0.000±0.000 a | 0.000±0.000 a | 0.000±0.000 a | 0.000±0.000 a | 0.000±0.000 a | ns |
| 15ADON (mg/kg) |  |  |  |  |  |  |  |
| C-1 |  | 0.317±0.317 b | 0.121±0.091 b | 3.099±0.902 a | 5.900±1.069 a | 5.779±1.603 a | 0.276 |
| 1-2 |  | 0.028±0.014 c | 0.022±0.017 c | 3.026±0.911 b | 8.033±1.131 a | 4.454±1.183 b | 0.225 |
| 2-3 |  | 0.006±0.006 c | 0.007±0.004 c | 1.157±0.651 b | 2.241±0.540 a | 2.391±0.156 a | 0.199 |
| 3-4 |  | 0.000±0.000 b | 0.005±0.005 b | 0.086±0.086 b | 0.238±0.001 ab | 0.741±0.389 a | 0.162 |
| 4-H |  | 0.000±0.000 b | 0.013±0.013 b | 0.065±0.040 ab | 0.084±0.052 ab | 0.351±0.246 a | 0.112 |
| Rachis |  | 0.000±0.000 a | 0.000±0.000 a | 0.062±0.053 a | 0.019±0.005 a | 0.072±0.048 a | 0.040 |
| Husk |  | 0.043±0.004 a | 0.037±0.006 a | 0.119±0.065 a | 0.091±0.013 a | 0.083±0.021 a | 0.038 |
| Grains |  | 0.000±0.000 a | 0.000±0.000 a | 0.000±0.000 a | 0.000±0.000 a | 0.000±0.000 a | ns |

^y^ At maturity, FCR disease severity at the stem base was visually assessed using a 0 to 4 classes, where 0 = no visible lesions, 1 = brown at the point of tiller attachment and up to the first internode; 2 = brown up to the second internode; 3 = brown up to the third internode; and 4 = brown up to the fourth internode. The stems (with the leaf sheath removed) were divided into eight segments to produce the tissue samples: C-1, first internode; 1-2, second internode; 2-3, third internode; 3-4, fourth internode; 4-H, penducle; rachis; husk; and grain.

^Z^ The 0.000±0.000 indicated not dectected. The detection limits of toxins were in wheat 10 µg/kg for DON and NIV, 10 µg/kg for 3ADON, 3 µg/kg for 15ADON, and 1 µg/kg for D3G. The quantitative analysis were performed in an Ultimate 3000 ultrahigh performance liquid chromatography coupled with Q Exactive-Orbitrap High Resolution Mass Spectrometer (Thermo Fisher Scientific, USA). The limit of detection of *F. pseudograminearum* DNA was 3 pg. Means followed by the same letter within a row are not significantly different at *P* = 0.05 according to Fisher’s least significant difference (LSD); ns= not significant. To ensure homogeneity of variance, transformations were applied to isolation frequency (arcsine square root), *F. pseudograiminearum* biomass and toxin content (log(*x*+1)). Comparisons between isolation frequency, *F. pseudograminearum* biomass, and toxin content of the same position in different disease classes were performed by analyzing the mutiple comparisons Fisher’s least significant difference test in R (4.0.0). NIV was not detected.

^X^ DON = deoxynivalenol, its derivatives (D3G = DON-3-glucoside, 3ADON = 3-acetyldeoxynivalenol, 15ADON = 15-acetyldeoxynivalenol, and NIV = nivalenol), and ZEN = zearalenone.
