# Supplementary material for: Fusarium pseudograminearum biomass and toxin accumulation in wheat tissues with and without Fusarium crown rot symptoms
Source: Front Plant Sci. 2024 May 21;15:1356723. doi: 10.3389/fpls.2024.1356723 (PMC11148387; doi:10.3389/fpls.2024.1356723)
Supplement: Supplementary file 6 [file Table_3.docx]

Table S3. The isolation frequency of *Fusarium pseudograminearum*, *Fusarium pseudograminearum* biomass (Fpg DNA ng/mg), DON (mg/kg), D3G content (mg/kg), ZEN (mg/kg), 3ADON (mg/kg), 15ADON (mg/kg), and NIV (mg/kg) in different tissues of cv. 'Bainong 207' in Neihuang County, Anyang City, Henan Province in 2020 under different classes of Fusarium crown rot.

|  |  | Disease severity classes for tillers^y^ | | | | | | | | |  |
| --- | --- | --- | --- | --- | --- | --- | --- | --- | --- | --- | --- |
| Characteristic^z^ |  | Class 0 | Class 1 | | Class 2 | | Class 3 | | Class 4 | | LSD_0.05_ |
| isolation frequency% |  |  | |  | |  | |  | |  |  |
| C-1 |  | 29.6±9.8 d | | 40.7±16.1 cd | | 66.7±0.0 bc | | 100±0.0 a | | 74.1±9.8 b | 19.5 |
| 1-2 |  | 0.0±0.0 d | | 0.0±0.0 d | | 33.3±12.8 c | | 70.4±3.7 b | | 92.6±3.7 a | 15.2 |
| 2-3 |  | 0.0±0.0 b | | 3.7±3.7 b | | 3.7±3.7 b | | 40.7±7.4 a | | 70.4±16.1 a | 24.8 |
| 3-4 |  | 0.0±0.0 b | | 0.0±0.0 b | | 3.7±3.7 ab | | 22.2±12.8 ab | | 44.4±25.7 a | 34.9 |
| 4-H |  | 0.0±0.0 a | | 0.0±0.0 a | | 3.7±3.7 a | | 3.7±3.7 a | | 3.7±3.7 a | 15.8 |
| Rachis |  | 0.0±0.0 a | | 0.0±0.0 a | | 0.0±0.0 a | | 0.0±0.0 a | | 0.0±0.0 a | ns |
| Husk |  | 0.0±0.0 a | | 0.0±0.0 a | | 0.0±0.0 a | | 0.0±0.0 a | | 0.0±0.0 a | ns |
| Grains |  | 0.0±0.0 a | | 0.0±0.0 a | | 0.0±0.0 a | | 0.0±0.0 a | | 0.0±0.0 a | ns |
| DNA (Fpg DNA ng/mg) |  |  | |  | |  | |  | |  |  |
| C-1 |  | 2.015±0.856 b | | 26.234±8.733 ab | | 47.956±33.903 ab | | 35.188±18.972 ab | | 149.410±31.565 a | 1.234 |
| 1-2 |  | 0.387±0.220 d | | 5.737±1.771 c | | 29.472±5.998 b | | 44.503±8.323 ab | | 77.127±3.754 a | 0.275 |
| 2-3 |  | 0.006±0.002 c | | 1.404±0.939 c | | 24.768±5.173 b | | 40.743±11.979 b | | 147.338±23.642 a | 0.340 |
| 3-4 |  | 0.006±0.001 c | | 0.030±0.016 c | | 9.507±4.324 b | | 9.781±3.452 b | | 78.051±13.653 a | 0.429 |
| 4-H |  | 0.005±0.001 a | | 0.011±0.005 a | | 0.057±0.033 a | | 0.296±0.287 a | | 0.315±0.151 a | 0.145 |
| Rachis |  | 0.000±0.000 b | | 0.003±0.003 b | | 0.023±0.017 b | | 0.033±0.020 b | | 0.443±0.224 a | 0.099 |
| Husk |  | 0.000±0.000 c | | 0.000±0.000 c | | 0.000±0.000 c | | 0.010±0.002 b | | 0.034±0.003 a | 0.002 |
| Grains |  | 0.000±0.000 a | | 0.000±0.000 a | | 0.000±0.000 a | | 0.000±0.000 a | | 0.000±0.000 a | ns |
| DON (mg/kg)^x^ |  |  | |  | |  | |  | |  |  |
| C-1 |  | 0.448±0.448 d | | 7.055±2.541 c | | 28.433±2.544 b | | 91.406±9.63 a | | 106.757±3.722 a | 0.323 |
| 1-2 |  | 0.000±0.000 c | | 0.827±0.827 c | | 22.541±3.831 b | | 49.561±4.007 a | | 92.944±9.089 a | 0.283 |
| 2-3 |  | 0.000±0.000 d | | 0.000±0.000 d | | 6.946±1.411 c | | 30.106±4.004 b | | 42.460±1.275 a | 0.144 |
| 3-4 |  | 0.000±0.000 c | | 0.000±0.000 c | | 1.263±0.639 b | | 3.429±0.527 a | | 3.423±0.496 a | 0.239 |
| 4-H |  | 0.000±0.000 b | | 0.000±0.000 b | | 2.25±0.786 a | | 3.231±0.676 a | | 3.331±0.676 a | 0.198 |
| Rachis |  | 0.000±0.000 c | | 0.000±0.000 c | | 3.166±0.769 b | | 8.673±1.473 a | | 4.190±0.831 b | 0.190 |
| Husk |  | 0.000±0.000 c | | 0.000±0.000 c | | 2.943±0.586 b | | 5.425±0.609 a | | 5.518±0.213 a | 0.118 |
| Grains |  | 0.000±0.000 a | | 0.000±0.000 a | | 0.000±0.000 a | | 0.000±0.000 a | | 0.000±0.000 a |  |
| D3G (mg/kg) |  |  | |  | |  | |  | |  |  |
| C-1 |  | 1.467±1.467 c | | 15.346±5.102 b | | 57.586±10.482 a | | 85.245±6.209 a | | 45.998±5.296 a | 0.450 |
| 1-2 |  | 0.735±0.735 c | | 6.609±2.636 b | | 41.607±10.592 a | | 88.299±10.391 a | | 107.245±13.853 a | 0.425 |
| 2-3 |  | 0.000±0.000 d | | 4.033±1.457 c | | 38.240±3.967 b | | 94.393±16.659 a | | 151.196±4.622 a | 0.260 |
| 3-4 |  | 0.000±0.000 d | | 1.741±0.402 c | | 22.304±4.237 b | | 64.824±9.610 a | | 91.285±7.062 a | 0.172 |
| 4-H |  | 0.000±0.000 d | | 2.352±0.797 c | | 23.058±3.645 b | | 56.276±3.859 a | | 72.777±1.104 a | 0.173 |
| Rachis |  | 0.000±0.000 d | | 2.614±0.545 c | | 14.245±2.245 b | | 45.849±2.784 a | | 39.890±0.564 a | 0.139 |
| Husk |  | 0.000±0.000 d | | 2.055±0.667 c | | 10.087±1.758 b | | 25.210±2.936 a | | 18.047±0.682 a | 0.200 |
| Grains |  | 0.000±0.000 b | | 0.000±0.000 b | | 0.000±0.000 b | | 0.053±0.028 a | | 0.086±0.009 a | 0.017 |
| ZEN (mg/kg) |  |  | |  | |  | |  | |  |  |
| C-1 |  | 0.000±0.000 c | | 0.055±0.055 c | | 1.250±0.830 c | | 5.218±1.788 b | | 26.869±0.559 a | 0.298 |
| 1-2 |  | 0.000±0.000 a | | 3.274±3.233 a | | 0.298±0.231 a | | 0.284±0.051 a | | 0.828±0.426 a | 0.510 |
| 2-3 |  | 0.000±0.000 a | | 0.000±0.000a | | 0.000±0.000 a | | 0.000±0.000 a | | 0.022±0.022 a | 0.013 |
| 3-4 |  | 0.000±0.000 a | | 0.000±0.000 a | | 0.000±0.000 a | | 0.000±0.000 a | | 0.000±0.000 a | ns |
| 4-H |  | 0.000±0.000 a | | 0.000±0.000 a | | 0.000±0.000 a | | 0.000±0.000 a | | 0.000±0.000 a | ns |
| Rachis |  | 0.000±0.000 a | | 0.000±0.000 a | | 0.000±0.000 a | | 0.000±0.000 a | | 0.000±0.000 a | ns |
| Husk |  | 0.000±0.000 a | | 0.000±0.000 a | | 0.000±0.000 a | | 0.000±0.000 a | | 0.000±0.000 a | ns |
| Grains |  | 0.000±0.000 a | | 0.000±0.000 a | | 0.000±0.000 a | | 0.000±0.000 a | | 0.000±0.000 a | ns |
| 3ADON (mg/kg) |  |  | |  | |  | |  | |  |  |
| C-1 |  | 0.000±0.000 b | | 0.000±0.000 b | | 0.000±0.000 b | | 2.190±0.369 a | | 2.459±0.526 a | 0.115 |
| 1-2 |  | 0.000±0.000 c | | 0.000±0.000 c | | 0.000±0.000 c | | 1.052±0.288 b | | 1.757±0.178 a | 0.098 |
| 2-3 |  | 0.000±0.000 b | | 0.000±0.000 b | | 0.000±0.000 b | | 0.831±0.538 ab | | 1.091±0.559 a | 0.271 |
| 3-4 |  | 0.000±0.000 a | | 0.000±0.000 a | | 0.000±0.000 a | | 0.000±0.000 a | | 0.000±0.000 a | ns |
| 4-H |  | 0.000±0.000 a | | 0.000±0.000 a | | 0.000±0.000 a | | 0.000±0.000 a | | 0.000±0.000 a | ns |
| Rachis |  | 0.000±0.000 a | | 0.000±0.000 a | | 0.000±0.000 a | | 0.000±0.000 a | | 0.000±0.000 a | ns |
| Husk |  | 0.000±0.000 a | | 0.000±0.000 a | | 0.000±0.000 a | | 0.000±0.000 a | | 0.000±0.000 a | ns |
| Grains |  | 0.000±0.000 a | | 0.000±0.000 a | | 0.000±0.000 a | | 0.000±0.000 a | | 0.000±0.000 a | ns |

^y^ At maturity, FCR disease severity at the stem base was visually assessed using a 0 to 4 classes, where 0 = no visible lesions, 1 = brown at the point of tiller attachment and up to the first internode; 2 = brown up to the second internode; 3 = brown up to the third internode; and 4 = brown up to the fourth internode. The stems (with the leaf sheath removed) were divided into eight segments to produce the tissue samples: C-1, first internode; 1-2, second internode; 2-3, third internode; 3-4, fourth internode; 4-H, penducle; rachis; husk; and grain.

^Z^ The 0.000±0.000 indicated not dectected. The detection limits of toxins were in wheat 10 µg/kg for DON and NIV, 10 µg/kg for 3ADON, 3 µg/kg for 15ADON, and 1 µg/kg for D3G. The quantitative analysis were performed in an Ultimate 3000 ultrahigh performance liquid chromatography coupled with Q Exactive-Orbitrap High Resolution Mass Spectrometer (Thermo Fisher Scientific, USA). The limit of detection of *F. pseudograminearum* DNA was 3 pg. Means followed by the same letter within a row are not significantly different at *P* = 0.05 according to Fisher’s least significant difference (LSD); ns= not significant. To ensure homogeneity of variance, transformations were applied to isolation frequency (arcsine square root), *F. pseudograiminearum* biomass and toxin content (log(*x*+1)). Comparisons between isolation frequency, *F. pseudograminearum* biomass, and toxin content of the same position in different disease classes were performed by analyzing the mutiple comparisons Fisher’s least significant difference test in R (4.0.0). NIV and 15ADON were not detected.

^X^ DON = deoxynivalenol, its derivatives (D3G = DON-3-glucoside, 3ADON = 3-acetyldeoxynivalenol, 15ADON = 15-acetyldeoxynivalenol, and NIV = nivalenol), and ZEN = zearalenone.
